# Automated collective motion analysis validates human keratinocyte stem cell cultures

**DOI:** 10.1038/s41598-019-55279-4

**Published:** 2019-12-10

**Authors:** Koji Kinoshita, Takuya Munesue, Fujio Toki, Masaharu Isshiki, Shigeki Higashiyama, Yann Barrandon, Emi K. Nishimura, Yoshio Yanagihara, Daisuke Nanba

**Affiliations:** 1 Graduate School of Science and Engineering, Ehime University, 3 Bunkyo-cho, Matsuyama, Ehime 790-8577 Japan; 2 Department of Stem Cell Biology, Medical Research Institute, Tokyo Medical and Dental University, 1-5-45, Yushima, Bunkyo-ku, Tokyo, 113-8510 Japan; 3 Division of Cell Growth and Tumor Regulation, Proteo-Science Center, Ehime University, Shitsukawa, Toon, Ehime, 791-0295 Japan; 4 Department of Biochemistry and Molecular Genetics, Ehime University Graduate School of Medicine, Toon, Shitsukawa, Ehime, 791-0295 Japan; 5 Institute of Medical Biology, A*STAR, Duke-NUS Graduate Medical School, and Department of Plastic, Reconstructive and Aesthetic Surgery, Singapore General Hospital, Singapore

**Keywords:** Time-lapse imaging, Skin stem cells

## Abstract

Identification and quality assurance of stem cells cultured in heterogeneous cell populations are indispensable for successful stem cell therapy. Here we present an image-processing pipeline for automated identification and quality assessment of human keratinocyte stem cells. When cultivated under appropriate conditions, human epidermal keratinocyte stem cells give rise to colonies and exhibit higher locomotive capacity as well as significant proliferative potential. Image processing and kernel density estimation were used to automatically extract the area of keratinocyte colonies from phase-contrast images of cultures containing feeder cells. The DeepFlow algorithm was then used to calculate locomotion speed of the colony area by analyzing serial images. This image-processing pipeline successfully identified keratinocyte stem cell colonies by measuring cell locomotion speed, and also assessed the effect of oligotrophic culture conditions and chemical inhibitors on keratinocyte behavior. Therefore, this study provides automated procedures for image-based quality control of stem cell cultures and high-throughput screening of small molecules targeting stem cells.

## Introduction

Adult stem cells in self-renewing tissues, such as the epidermis and corneal epithelium, can be cultivated and massively expanded under appropriate conditions. When co-cultured with lethally-irradiated mouse 3T3 fibroblasts, human epidermal keratinocyte stem cells can maintain themselves, give rise to differentiated progeny, and become organized into squamous epithelial structures *ex vivo*^1,2^. This has allowed autologous transplantation of cultured keratinocyte sheets onto burn patients^3,4^; the first successful application of cultured human stem cells isolated from patients for regenerative medicine^5–7^. This technology has since been adapted to cell therapy for ocular surface reconstruction^8–11^ and autologous transplantation of genetically modified epidermal sheets^12–15^.

Autologous transplantation of cultured keratinocytes has long been successfully applied to treat patients with severe burns and corneal blindness. However, the transplantation can fail if stem cells are lost from the culture, even if the generated sheets are engrafted successfully^16–18^. Keratinocyte stem cells can lose their stem cell properties during serial cultivation and/or under suboptimal culture conditions, and transform into cells with more restricted potential. This phenomenon is termed “clonal conversion”, and results in progressive and irreversible reduction of the proliferative potential of keratinocytes, which makes the cultures inappropriate for transplantation^19,20^. Thus, *ex vivo* maintenance and expansion of keratinocyte stem cells are essential to the success of cell therapy, and the evaluation of stem cell cultures is required for advancing stem cell-based regenerative medicine. However, the quality assurance of stem cell cultures currently depends on individual cell culture experts, therefore cannot be standardized.

Image-based identification of cultured stem cells and noninvasive evaluation of their potency could provide standardized quality assurance of stem cell cultures required for regenerative medicine, and may also allow high-throughput screening in stem cell research^21,22^. Several studies have reported that the potency of human mesenchymal stem cells and pluripotent stem cells can be noninvasively evaluated by analyzing cell or colony morphology^23–26^. Time-lapse imaging has also been used to estimate the potency of human stem cells^27,28^. Recently, we revealed that human epidermal keratinocyte stem cell colonies exhibit a higher locomotive phenotype^29^. Our study demonstrated that human keratinocyte stem cells can be identified by image analysis of cell movement, giving the basis of a noninvasive method to estimate the growth potential of keratinocytes in culture and monitor and validate the quality of cells for transplantation. However, human keratinocytes give rise to densely packed colonies, and the motion of individual cells within the colony has so far been analyzed by manual cell tracking. This process is extremely time-consuming, laborious, and at risk of human error. Furthermore, the human keratinocyte culture requires feeder cells, which complicates the automatic identification of keratinocytes among the heterogeneous cell population. The feeder cell-based culture system has also been adapted to other stem cell cultures. Therefore, applying our findings to cell manufacturing for regenerative medicine will require automation of both the identification of human keratinocyte colonies among heterogeneous cell populations, and the estimation of cell motion speed within the colony.

Here we provide an image-processing pipeline that noninvasively identifies human keratinocyte stem cells and validates the quality of human keratinocyte cultures for transplantation. This pipeline consists of two main modules: the identification of human keratinocyte colonies on the feeder layer of 3T3 cells, and the estimation of cell locomotion speed using optical flow.

## Methods

### Cell culture

Normal human epidermal keratinocytes were isolated from neonatal skin. Frozen keratinocytes were thawed and cultivated at clonal density on a feeder layer of irradiated or mitomycin C-treated mouse 3T3-J2 fibroblasts. The co-culture was incubated at 37 °C with 10% CO_2_ in a 3:1 mixture of Dulbecco-Vogt modification of Eagle’s medium (DMEM) and Ham’s F12 medium, supplemented with 10% fetal bovine serum (FBS), 1.8 × 10^−4^ M adenine hemisulfate salt, 5 μg/ml insulin, 0.4 μg/ml hydrocortisone, 10^−10^ M cholera toxin, and 2 × 10^−9^ M triiodothyronine (T3), as described previously^1,30^. Keratinocytes between passages 2 and 7 were used for experiments, and the medium was changed every 4 days. Pharmacological inhibitors used for the analysis of cell locomotion were purchased from Wako (Y-27632 and cytochalasin D) and Sigma–Aldrich (2-deoxy-D-glucose and (-)-blebbistatin). Small molecule inhibitors were added 1 h before collecting images.

### Time-lapse imaging

Human epidermal keratinocytes were seeded at clonal density in a 35 mm cell culture dish (Corning) with irradiated or mitomycin C-treated 3T3-J2 cells, and grown for 6 or 7 days in the presence of EGF. For time-lapse imaging, cells were maintained at 37 °C with 10% CO_2_, in a chamber mounted on an Axiovert 200 M microscope (ZEISS). Images were obtained at 5 min intervals for 5, 10, or 180 min, depending on the experimental conditions, with an AxioCamHR3 monochrome camera (ZEISS) using EC Plan-Neofluar 5×/0.16 and 10×/0.30, and LD A-Plan 20×/0.30 objective lenses.

### Extraction of human keratinocyte colony area

An Open Source Computer Vision Library (OpenCV), an open source computer vision and machine learning software library, is used for the image processing in this study. Extraction of human keratinocyte colony area was constructed by three procedures: (1) extracting the center of cells, (2) extracting branch points of the cell boundary, (3) using kernel density estimation to evaluate the likelihood of pixels belonging to a colony.

(1) Extracting the center of cells: First, we applied adaptive binarization^31^ to grayscale images in order to extract the nucleoli, which are recognized as dark spots, and the nucleus, which is recognized as a dark ring. The threshold value is a weighted average of the neighborhood of processed pixels. In this study, we set a 41 × 41 pixel region as the neighborhood, then extracted connected components and remaining regions of ≥12 pixels or ≤72 pixels. The centroid of each remaining region was then calculated. The centroid was defined as the nucleoli, which had to be located in its own region and also surrounded by the ring-shaped region (nucleus).

(2) Extracting branch points of cell boundary: First, we applied adaptive thresholding^31^ to grayscale image in order to extract cell-cell boundary. Then, we applied opening and closing operation to eliminate small spots or protrusions. Then, we removed small regions which have 250 pixels or less by using labeling algorithm. Then we extracted skeleton image by Zhang-Suen thinning algorithm^32^. Finally, the branching points were extracted by the Crossing Number (CN) method^33^. This method extracts the bifurcations from the skeleton image by examining each 8-neighbor pixels ${P}_{i}\in \{0,\,1\}\,(i=1,\cdots ,\,8)$ using a 3 × 3 window including target pixel *P*. *P*_*i*_ = 1 is skeleton pixel of cell-cell boundary, *P*_*i*_ = 0 is background pixel. The CN is defined by

$$CN=0.5\mathop{\sum }\limits_{i=1}^{8}|{P}_{i}-{P}_{i+1}|,\,{P}_{9}={P}_{1}$$

This number is difference of the adjacent pixel values. If CN is equal to 3, pixel is branching point.

(3) Using kernel density estimation to evaluate the likelihood of pixels belonging to a colony:

Kernel density estimation is a non-parametric method to estimate probability density function. Let (*x*_1_, *x*_2_ *x*_*n*_) be a sample points drawn from an unknown distribution *f*(*x*). We estimate the shape of this distribution as follows;

$$f(x)=\frac{1}{nh}\sum _{i}\,K(\frac{x-{x}_{i}}{h}),$$

where *K* is a non-negative kernel function and *h* > 0 is a smoothing parameter called bandwidth. The extracted center of cells by method in (1) and branching points by method in (2) are regarded as sample points *x*_*i*_ of kernel density estimation. There exists numerous center of cells and branching points in colony region, hence the estimated distribution *f*(*x*) expresses the likelihood that a pixel *x* belongs to a colony. In this study, we used the Gaussian kernel and set the bandwidth to *h* = 151. Pixels with a high likelihood value were regarded as belonging to a colony. Hence, we applied thresholding to the estimated distribution and extracted the largest region as the colony.

### Measurement of optical flow

Optical flow is the pattern of apparent velocities of movement of a particular brightness pattern in an image. In this study, we calculated optical flow using the DeepFlow algorithm^34^, which is based on a deep, multi-layer, convolutional architecture. The DeepFlow module was implemented in the OpenCV image processing tool, with the parameters set at default values. We measured the average magnitude of the estimated optical flow in each extracted colony region.

### Statistical analysis

Prism8 software (GraphPad Software) was used to assess statistical significance. To determine significance between two groups, comparisons were performed using an unpaired two-tailed Mann-Whitney *U*-test. For multiple comparisons, Kruskal-Wallis test with Dunn’s post hoc test were performed. *P* < 0.05 was considered statistically significant.

## Results

### An image-processing pipeline for collective motion analysis of human keratinocyte colonies

**Figure 1:**
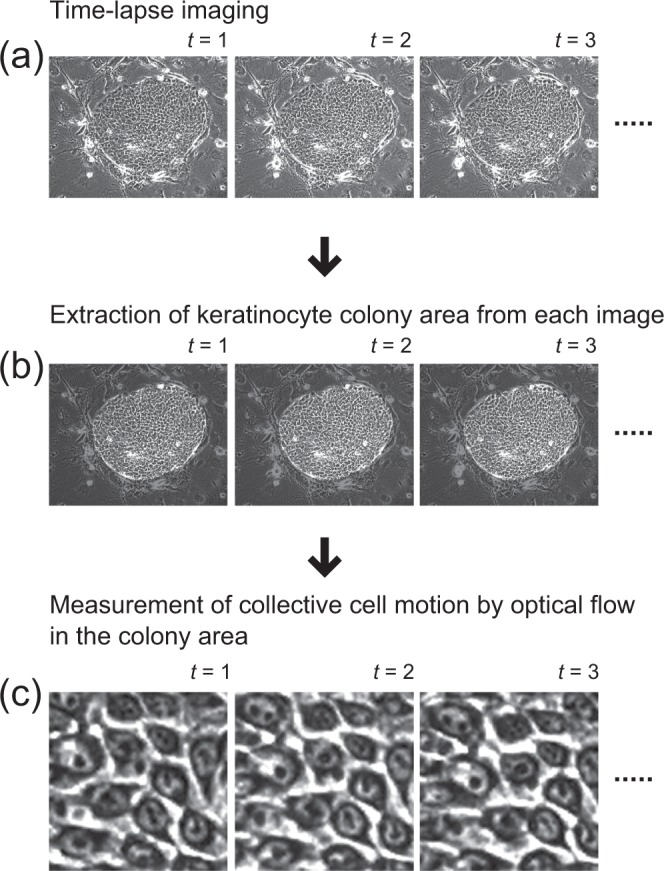
An image-processing pipeline for collective motion analysis of human keratinocyte colonies. (**a**) Acquisition of time-series phase-contrast images of human keratinocyte colonies. In this study, images were obtained at 5 min intervals. (**b**) Extraction of keratinocyte colony area from a set of time-series phase-contrast images. (**c**) Measurement of collective cell motion by optical flow in the extracted colony area.

We hypothesized that automated collective motion analysis in human keratinocyte stem cell cultures could be developed as follows: (1) Obtain time-series phase-contrast images of human keratinocyte colonies by time-lapse imaging of a culture containing both keratinocyte colonies and inactivated mouse 3T3 fibroblasts (Fig. 1a). (2) Extract the area of each keratinocyte colony from the time-series images in order to analyze the collective motion of keratinocytes (Fig. 1b). (3) In the extracted areas, visualize the motion of each pixel by optical flow, allowing the locomotion speed of cells to be estimated (Fig. 1c).

### Image-processing for identification of keratinocyte colonies in heterogeneous cultures

**Figure 2:**
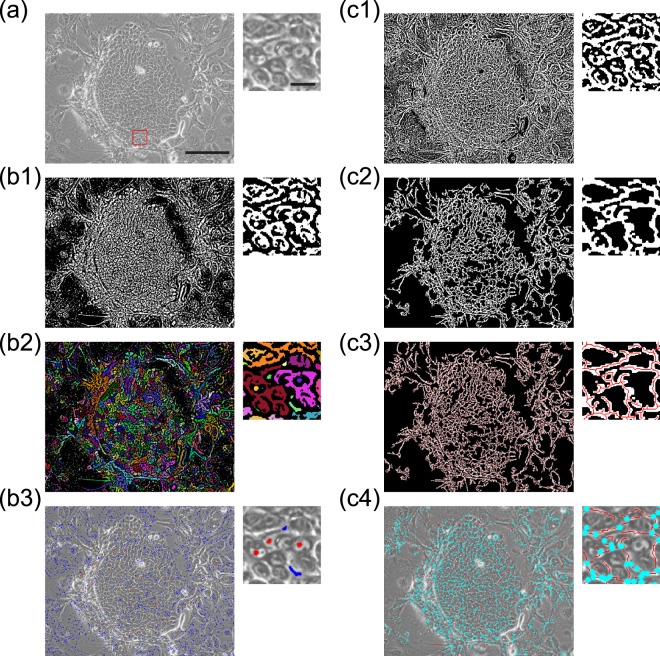
Two image-processing procedures for identification of keratinocyte colonies. (**a**) An original phase-contrast image before processing. The red-boxed area in the left panel is shown at a higher magnification in the right panel. Bars, 100 μm (left) and 10 μm (right). (**b1-b3**) Identification of nucleoli in the keratinocytes. The images from A following each image-processing procedure are shown, including the magnified view (right panels). After adaptive binarization to highlight the nucleus in the keratinocytes (b1), each contiguous region was labeled with pseudo color (b2). The centroid of each contiguous region was defined as the nucleolus of cells (red spots), providing that this was within the contiguous region itself (b3). The blue areas are also the central continuous regions, but not defined as the nucleoli because they are not surrounded by the second contiguous regions. See also Methods and Supplementary Fig. 1. (**c1-c4**) Identification of cell-cell boundaries in the keratinocytes. The images from A following each image-processing procedure are shown, including the magnified view (right panels). Another type of adaptive binarization was performed to highlight cell-cell boundaries in the keratinocyte colonies (c1). The highlighted area was flattened by noise reduction (c2), and thinned for extraction of cell-cell boundaries as lines (c3). As keratinocytes in the colony are surrounded by several neighbors, the lines produce branching points (blue spots) (c4).

We developed two image-processing procedures to extract keratinocyte colony areas from phase-contrast images of keratinocytes cultured with 3T3 feeder cells. Human keratinocytes, including stem cells, have significant proliferative capacity and give rise to progressively growing colonies when cultivated under appropriate conditions^2,20^. These growing colonies consist of densely packed small cells, making it difficult to distinguish individual keratinocytes within the colony in phase-contrast images. However, the nucleus of each keratinocyte can be easily identified and used for manual cell tracking^29,35^. In phase-contrast images, the nucleoli are recognized as dark spots within the nucleus of cells, and the nucleus itself is recognized as a dark ring at its boundary with the cytoplasm (Fig. 2a). This gradient of brightness can be clarified using adaptive binarization. Using this image processing technology, we first highlighted the dark regions (the nucleoli and cytoplasm around the nucleus) as the binary image (Fig. 2b1), then labeled the contiguous regions of the highlighted areas with pseudo colors (Fig. 2b2). Next, we defined the centroid of the contiguous region as the nucleolus (Fig. 2b3), with the condition that it must be within the central contiguous region (nucleolus), and also surrounded by the second contiguous region (nuclear boundary) (see details in Methods and Supplementary Fig. 1).

Human keratinocyte colonies are characterized by the aggregation of cells, and the boundary of cells in the colony can be identified as bright regions in phase-contrast images (Fig. 2a). Using this feature, we developed another image processing procedure to distinguish the keratinocyte colonies from the feeder cells. Adaptive binarization was employed to highlight the bright regions at the cell-cell boundaries (Fig. 2c1). The highlighted area was then flattened using noise reduction (Fig. 2c2), and labeled by thinning of the contiguous region. This image-processing procedure allowed the cell-cell boundary to be extracted as a line (Fig. 2c3). For individual cells surrounded by several neighbors, the lines of the cell boundaries form a mesh structure, which includes branching points. Therefore, the whole area of a keratinocyte colony can be identified as containing multiple branching points as shown in blue (Fig. 2c4) (see details in Methods and Supplementary Fig. 2).

### Extraction of keratinocyte colony area using kernel density estimation

**Figure 3:**
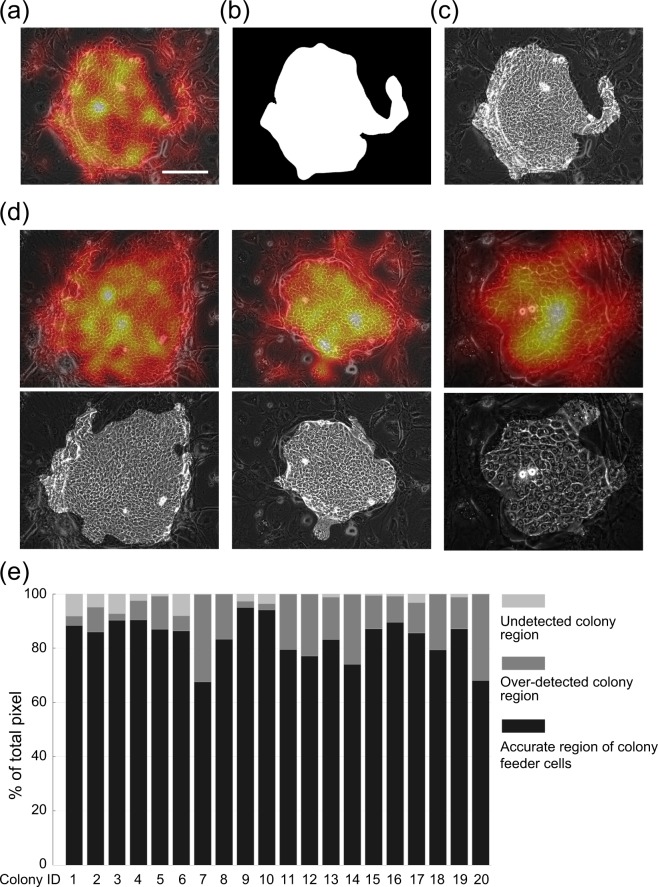
Extraction of keratinocyte colony area using kernel density estimation. (**a**) Density mapping on a phase-contrast image of a human keratinocyte colony with feeder cells. The density of the nucleoli and branching points identified in Fig. 2 is indicated by the degree of colors. Bar, 100 μm. (**b**) Areas where the density of nucleoli and branching points was above a certain threshold were extracted using kernel density estimation. (**c**) Extraction of the area containing a high density of nucleoli and branching points from a phase-contrast image of a human keratinocyte colony with feeder cells. (**d**) Three examples of human keratinocyte colony extraction from phase-contrast images of areas containing feeder cells. (**e**) Evaluation of the human keratinocyte colony extraction using kernel density estimation. The automatically extracted areas were compared with the keratinocyte colony areas defined by manual extraction. Evaluation values are defined as follows. True positive (TP) is number of pixels correctly extracted as the colony region. False positive (FP) is number of pixels extracted as the colony automatically but defined as the feeder cells manually. False negative (FN) is number of pixels extracted as feeder cells automatically but defined as the colony region manually. True negative (TN) is number of pixels correctly extracted as the feeder cells. Undetected colony region means ratio of FN towards total pixels, FN / (TP + FP + FN + TN). Over-detected colony region means ratio of FP toward to total pixels, FP/(TP + FP + FN + TN). Accurate region of colony and feeder cells means ratio of TP and TN toward to total pixels, (TP + TN)/(TP + FP + FN + TN).

The area of the keratinocyte colonies contains a high density of cell nucleoli and cell-cell boundary branching points (Fig. 2). Therefore, we were able to extract the keratinocyte colony area using kernel density estimation (see details in Methods). In brief, an area was assumed to be a keratinocyte colony if the nucleoli and branching structures were both above a certain density (Fig. 3a,b). Although 3T3 cells were also occasionally recognized as keratinocyte colonies when their aggregation was continuous with the keratinocytes (Fig. 3c), this method was mainly successful in extracting the area of keratinocyte colonies (Fig. 3d,e).

### Estimation of cell locomotion speed by optical flow

**Figure 4:**
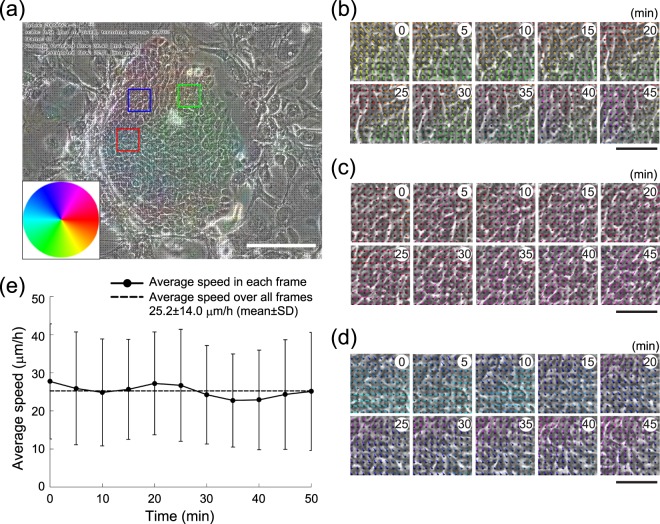
Estimation of cell locomotion speed by optical flow. (**a**) The area extracted using kernel density estimation (Fig. 3) was analyzed by optical flow to estimate cell locomotion speed. The colored circle indicates the direction of optical flow. Bar, 100 μm. (**b**–**d**) Magnified time-series images of the areas indicated by the boxes in a (b, green box; c, blue box; d, red box). The optical flow is indicated by colored bars. The colors also indicate the direction of optical flow as shown in A. Bars, 20 μm. (**e**) The average speed of cell locomotion in the extracted area was estimated by optical flow at 5 min intervals. The average speed of cells in the colony was consistent during the entire period of time-lapse imaging. Bars show standard deviations.

We next sought to apply estimation of cell movements in the extracted area. Time-series phase-contrast images of human keratinocyte colonies recorded at 5 min intervals were processed as described above, and the optical flow of the objects in the extracted area was subsequently analyzed using the DeepFlow algorithm (Fig. 4a and see details in Methods). The collective motion of keratinocytes at 5 min intervals was successfully traced using this procedure (Fig. 4b–d). We also calculated the average speed of cells in the colonies, which was largely consistent during each 5 min period, and throughout the entire period of time-lapse imaging (Fig. 4e).

### Automated collective motion analysis identifies human keratinocyte stem cells

**Figure 5:**
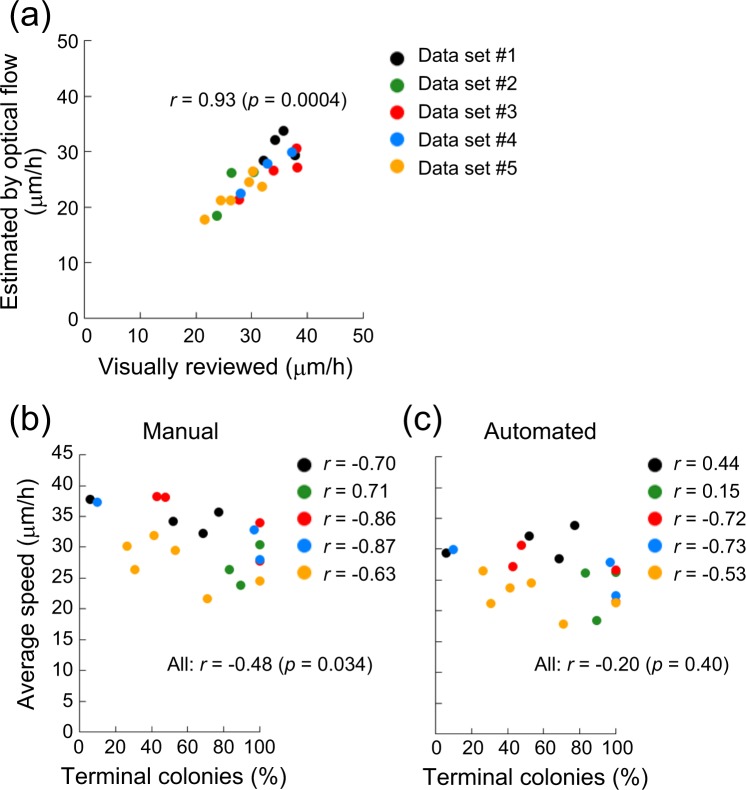
Automated collective motion analysis identifies human keratinocyte stem cell colonies. (**a**) Strong positive correlation of cell locomotion speed between manual cell tracking and automated estimation. The image data of human keratinocyte colonies that had already been analyzed with manual cell tracking^29^ were re-examined with the automated image-processing pipeline developed in this study. (**b**) Correlation between the incidence of terminal colonies after cloning and the average speed of cells analyzed with manual cell tracking as shown previously^29^. Each experiment, except for the experimental data set shown in green dots, revealed a negative correlation between the average speed of cells in the colony and the incidence of terminal colonies assessed by clonal analysis. (**c**) Correlation between the incidence of terminal colonies after cloning and the average speed of cells analyzed by the automated image-processing pipeline developed in this study. Only one experimental data set (shown in black dots) showed an opposite correlation to the analysis performed with manual cell tracking.

Having developed the image-processing pipeline for analyzing collective motion of cells in human keratinocyte colonies, we applied the pipeline to time-lapse images which had been manually analyzed in our previous report^29^. We first compared the average speed of cells estimated by optical flow in this study with that calculated by manual tracking in the previous study. Although the average speed of cells calculated by the automated pipeline tended to be lower than that obtained by manual tracking, there was a strong positive correlation between the speed calculated by the two independent procedures from the same time-series (Fig. 5a). In our previous study, the percentage of terminal colonies in the culture dish after passage of a human keratinocyte colony was negatively correlated with the average speed of cells in the keratinocyte colony before passage^29^. Terminal colonies result from the clonal conversion of human keratinocyte stem cells^19^, and their appearance is clearly linked with the loss of epidermal stemness^2,20,29^. We reanalyzed the data sets (#1–5) which previously showed the negative correlation of the average speed of cells with the percentage of terminal colonies (Fig. 5b), and compared this result with that obtained by our new automated pipeline. With the exception of data set #1, the correlation of the average speed of cells with the percentage of terminal colonies was maintained when the speed was calculated by DeepFlow algorithm (Fig. 5c). These results indicate that human keratinocyte stem cell colonies can be identified by measuring cell locomotion speed by a combination of automated colony area extraction and estimation of optical flow.

### Validation of human keratinocyte culture conditions with collective motion analysis

**Figure 6:**
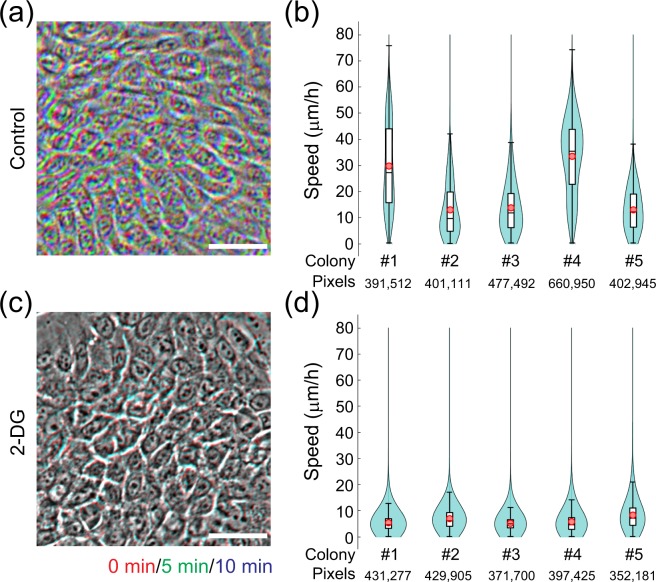
Validation of human keratinocyte culture conditions using collective motion analysis. (**a**) Collective motion of human keratinocytes within a colony. An overlay image of human keratinocytes at three different time-points, in red (0 min), green (5 min), and blue (10 min), under normal culture conditions is shown. The overlay produces a white/gray color if cell positions do not change over a short period of time. Bar, 50 μm. (**b**) Automated collective motion analysis of 5 human keratinocyte colonies in normal culture conditions using the image-processing pipeline developed in this study. Images taken at three time-points (5 min intervals for 10 min) were used to estimate cell locomotion speed. Violin plots indicate the variation of cell locomotion speed in each colony. (**c**) An overlay timecourse image (as described in a) of human keratinocytes under oligotrophic culture conditions with 50 mM 2-deoxy-D-glucose (2-DG). The white/gray image color of the overlay indicates very little cell movement under these conditions. Bar, 50 μm. (**d**) Violin plots of cell locomotion speed estimated by automated collective motion analysis for colonies under the conditions described in C. The oligotrophic culture conditions significantly reduced the locomotion of cells in the colony. *p* = 0.0079 vs. Control. P-value was calculated with average speed of each colony by Mann-Whitney *U*-test.

We next examined whether collective motion analysis could be applied for the quality control of human keratinocyte cultures. To reproduce oligotrophic culture conditions, we utilized 2-deoxy-D-glucose (2-DG), which inhibits glycolysis and depletes cellular stores of ATP. After the addition of 2-DG into the culture medium for 1 hr, we obtained 3 serial images of progressive growing colonies with 5 min intervals since the average speed of cells during 5 min is almost same as those during 60 min as shown in Fig. 4e. Manual analysis confirmed that the collective motion of keratinocytes was markedly inhibited by 2-DG treatment (Fig. 6a,c). The same time-series phase-contrast images were also analyzed with our image-processing pipeline, which confirmed that the automated collective motion analysis can quantitatively assess the collective motion of cells in keratinocyte colonies (Fig. 6b,d). This result indicates that our automated analysis can easily detect oligotrophic culture conditions in a short period of time, which strongly suggests that this system could be applied for quality assurance of human keratinocyte cultures for clinical applications and cell manufacturing.

### Short-term screening of the effect of small molecules on keratinocyte behavior

**Figure 7:**
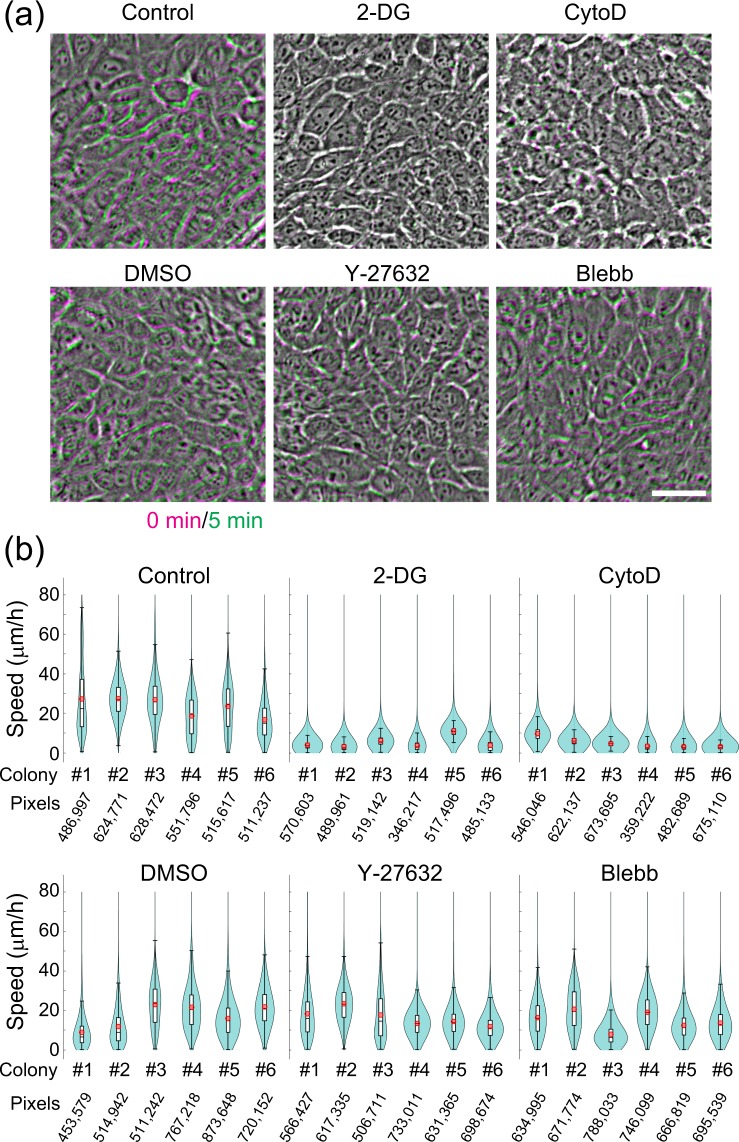
Short-term screening of the effect of small molecules on keratinocyte behavior using collective motion analysis. (**a**) Collective motion of human keratinocytes were examined following treatment with a variety of small molecules including 50 mM 2-DG, 1 μM cytochalasin D (CytoD), 10 μM Y-27632, and 10 μM blebbistatin (Blebb). The overlay of images at two different points, in magenta (0 min) and green (5 min), produces a white/gray color if cell positions do not change over a short period of time. Bar, 50 μm. (**b**) Violin plots of the cell locomotion speed estimated by automated collective motion analysis for colonies under the conditions described in A. The analysis reveals impairment of cell locomotion with 2-DG and CytoD using only two images obtained at 5 min intervals. *p* = 0.0004 (Control vs. 2-DG), *p* = 0.0002 (Cntorol vs. CytoD), *p* = 0.8970 (Control vs. DMSO), *p* = 0.7711 (Control vs. Y-27632), *p* = 0.4216 (Control vs. Blebb). P-values were calculated with average speed of each colony by Kruskal-Wallis test with Dunn’s multiple comparison test.

As our previous experiments revealed that only 2 or 3 serial images at 5 min intervals are sufficient for calculating the collective motion of cells in human keratinocyte colonies (Figs. 5, 6), we next examined whether collective motion analysis could be applied for short-term screening of small molecules targeting human keratinocytes. We first confirmed that automated motion analysis could detect inhibition of collective motion of keratinocytes by 2-DG using only 2 serial images at 5 min intervals (Fig. 7a,b). Disruption of actin polymerization by cytochalasin D (CytoD) also significantly reduced the collective motion of keratinocytes measured by the automated analysis (Fig. 7a,b). We previously reported that inhibition of actomyosin contractile force by Y-27632 (ROCK inhibitor) and blebbistatin (Blebb; myosin ATPase inhibitor) completely attenuates EGF-induced keratinocyte colony dynamics^20^. However, neither of these inhibitors interfered with collective motion in the colony (Fig. 7a,b). These results indicate that the automated collective motion analysis can quantitatively assess the effects of small molecules on human keratinocyte behavior over a short period of time. Collectively, this study provides strong evidence that automated collective motion analysis can be applied for image-based high-throughput screening of molecules targeting human keratinocytes, including stem cells.

## Discussion

Image-based identification of cultured human stem cells and noninvasive evaluation of their proliferative capacity has the potential to drastically advance stem cell-based regenerative medicine and stem cell research. Culture of human epidermal keratinocyte stem cells has been clinically applied for over three decades, yet reliable quality assurance methods have not been established. In this study, we developed an image-processing pipeline for noninvasive evaluation of human epidermal keratinocyte cultures, based on automated collective motion analysis. This image-processing pipeline confirmed that cell locomotion speed is linked with long-term proliferative capacity, and also detected short-term changes in cell locomotion due to oligotrophic culture conditions or treatment with chemical inhibitors.

In this study, we estimated cell locomotion speed by optical flow, which calculates the velocity vector of each individual pixel using its specific brightness information between time-series images. Optical flow has been applied in other areas of cell biology research, including intracellular transport, dynamics of subcellular structures, and cell motion^36^. In stem cell research, optical flow has been also used for analyzing the beat patterns of cardiomyocytes derived from human iPS cells^37^. Our study demonstrated that the average speed of keratinocytes within colonies measured by manual cell tracking strongly correlates with the cell locomotion speed estimated by optical flow. However, the values obtained by the optical flow method were consistently lower than those obtained by manual cell tracking. In addition, there was disagreement between the automated collective motion analysis and manual cell tracking results in one of the data sets. This suggests that, for reasons currently unknown, the principles of object and pixel tracking are not identical. Therefore, it may be necessary to develop an automated tracking system for objects in order to more precisely measure the locomotion speed of cells within colonies. Recent progress in deep learning-based methods might enable automated identification and tracking of single cells in the stem cell colony. However, our image processing method requires smaller number of image data than deep learning-based methods and can be easily applied in a variety of stem cell cultures.

This pipeline consists of two main modules: the identification of human keratinocyte colonies on the feeder layer of 3T3 cells, and the estimation of cell locomotion speed using optical flow. The murine fibroblast 3T3 cell line is indispensable for the massive expansion of human keratinocytes *ex vivo*^1,8^, and this co-culture system is required for cell therapy treatment of severe burns^3,4^ and corneal blindness^10,11,38^. Feeder-dependent cultures are also required for *ex vivo* maintenance and expansion of a variety of pluripotent and tissue-specific stem cells, which are essential for stem cell-based regenerative medicine. Noninvasive evaluation of stem cell properties by image analysis therefore requires the extraction of stem cell colonies from the feeder cells. Our method could also be applied in other stem cell culture systems, particularly those where the stem cells give rise to densely packed colonies. Recently, feeder-free cultures for stem cells, including human keratinocytes, with comparable properties to feeder-dependent cultures have been developed^39,40^, though their clinical use is currently limited. However, the colony area must be also extracted from images even in feeder-free cultures to estimate the locomotion speed of cells in the colony. Therefore, our system is applicable to both feeder-dependent large-scale stem cell cultures which are currently being used in the clinics and feeder-free stem cell cultures. Additionally, the image processing technology that we have developed for the extraction of keratinocyte colonies from feeder cells could be more widely applied for the identification of certain cell-types among heterogeneous cell populations.

Appropriate culture conditions are essential for the maintenance of stem cell properties, and the development of reliable real-time monitoring methods for stem cell cultures is crucial for cell manufacturing in regenerative medicine. Image-based stem cell culture monitoring is noninvasive, does not require labeling, and can be performed *in situ*. Although motion analysis takes more time than morphological analysis, we demonstrated that two serial images at 5 min intervals are sufficient to detect changes in cell locomotion under oligotrophic culture conditions and in the presence of chemical inhibitors. The time interval of images could be reduced even further if the conditions of image acquisition were better adapted for the motion analysis. This automated collective motion analysis could be applied for real-time monitoring of stem cell cultures, significantly advancing cell manufacturing for regenerative medicine.

## Supplementary information

Supplementary information
