# Supplementary material for: Automated collective motion analysis validates human keratinocyte stem cell cultures
Source: Sci Rep. 2019 Dec 10;9:18725. doi: 10.1038/s41598-019-55279-4 (PMC6904747; doi:10.1038/s41598-019-55279-4)
Supplement: Supplementary file 1 — Supplementary information [file 41598_2019_55279_MOESM1_ESM.pdf]

Supplementary Information

Automated collective motion analysis validates human keratinocyte stem cell cultures

Koji Kinoshita[1]*, Takuya Numesue[1], Fujio Toki[2], Masaharu Isshiki[1], Shigeki Higashiyama[3,4], Yann Barrandon[5], Emi K. Nishimura[2], Yoshio Yanagihara[1], and Daisuke Nanba[2]*

[1]Graduate School of Science and Engineering, Ehime University, 3 Bunkyo-cho, Matsuyama, Ehime 790-8577, Japan
[2]Department of Stem Cell Biology, Medical Research Institute, Tokyo Medical and Dental University, 1-5-45, Yushima, Bunkyo-ku, Tokyo 113-8510, Japan
[3]Division of Cell Growth and Tumor Regulation, Proteo-Science Center, Ehime University, Shitsukawa, Toon, Ehime 791-0295, Japan
[4]Department of Biochemistry and Molecular Genetics, Ehime University Graduate School of Medicine, Toon, Shitsukawa, Ehime 791-0295, Japan.
[5]Stem Cell Dynamics, Institute of Medical Biology, 8A Biomedical Grove, #06-06, Immunos, 138648, Singapore

Supplementary information

Supplementary Figure 1

Identification of nucleoli by image processing.

(a) Magnified view of a phase contrast image of the human keratinocyte colony shown in Fig. 2.

(b) After adaptive binarization, the centroid was defined as the nucleoli, providing that it was located in its own contiguous region and was also surrounded by the ring-shaped contiguous region.

Supplementary Figure 2

Identification of branching points by image processing.

Black Pixels are skeleton after applying Zhang-Suen thinning algorithm. We express target pixel as P (red rectangle) and 8-neighbor pixels P_i (blue rectangle). If the pixel P_i is skeleton then the value of pixel is assigned as $P_i = 1$. The crossing number is defined by counting difference of the adjacent pixel values. The crossing number in this case is equal to 3, hence, target pixel is branch points.

Supplementary Table 1

All numerical data analyzed in Figure 5.

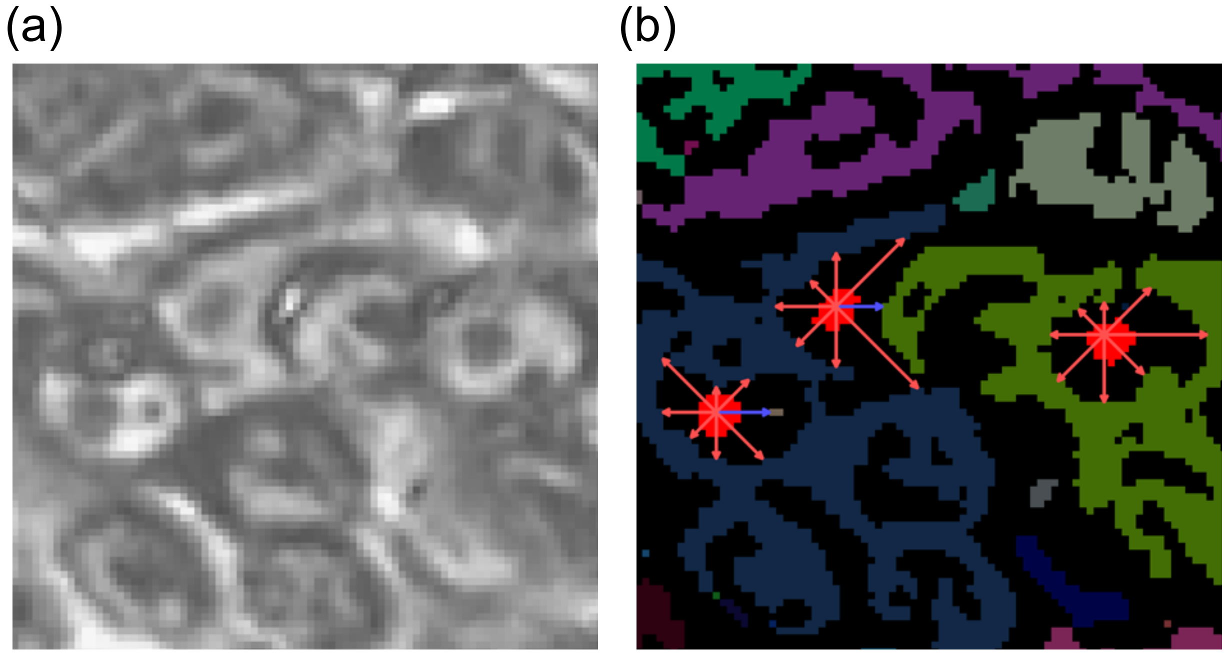
(a)
(b)

Supplementary Figure 2

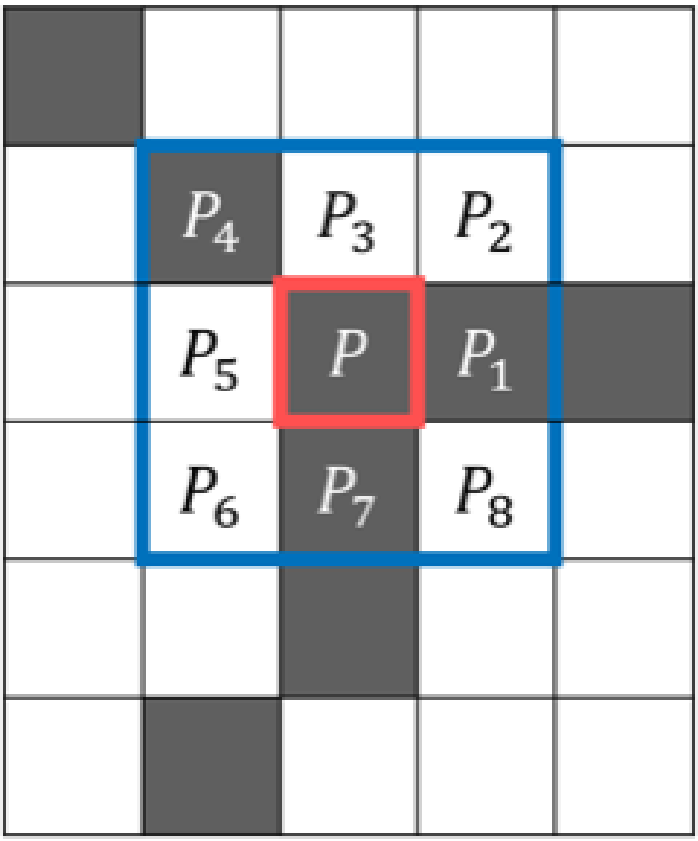

Supplementary Table 1

Colony ID	Terminal colonies (%)	Average speed (μm/h)	
		Manual	Automated
Data set #1			
20090924-2	68.7	32.3	25.2
20090924-3	77.2	35.7	32.4
20090924-5	5.6	37.8	25.0
20090924-6	52.1	34.2	33.2
Data set #2			
20091007-1	83.3	26.4	27.3
20091007-2	89.5	23.8	17.7
20091007-3	100.0	30.4	31.7
Data set #3			
20091014-2	100.0	33.9	29.0
20091014-3	100.0	27.7	20.8
20091014-4	43.0	38.2	25.2
20091014-5	47.7	38.1	32.7
Data set #4			
20091028-1	9.7	37.3	33.7
20091028-2	96.9	32.8	29.4
20091028-3	100.0	28.0	25.0
Data set #5			
20091111-1	100.0	24.5	23.2
20091111-2	71.1	21.6	18.1
20091111-3	26.3	30.2	26.7
20091111-4	30.6	26.3	23.9
20091111-5	41.2	31.9	25.4
20091111-6	53.3	29.5	27.1
